# The Frequency of Axial Deposition in Korean Patients With Gout at a Tertiary Spine Center

**DOI:** 10.3389/fmed.2020.00339

**Published:** 2020-08-05

**Authors:** Hyo-Joon Jin, Eun-Seok Son, Du Hwan Kim

**Affiliations:** ^1^Department of Rehabilitation Medicine, Dongsan Medical Center, School of Medicine, Keimyung University, Daegu, South Korea; ^2^Department of Orthopedic Surgery, Dongsan Medical Center, School of Medicine, Keimyung University, Daegu, South Korea; ^3^Department of Physical Medicine and Rehabilitation, College of Medicine, Chung-Ang University, Seoul, South Korea

**Keywords:** gout, spine, computed tomography, tophi, monosodium urate deposit

## Abstract

**Objectives:** This study aimed to describe the frequency of axial deposition (axial gout) and determine the associated factors in patients with gout who presented to a spine clinic in Korea.

**Methods:** We enrolled 95 Korean patients who visited our spine center from March 2012 to February 2016 and who had been previously diagnosed with gout and had available computed tomography (CT) images of the vertebral columns. Axial gout was defined as the presence of erosions or tophi in the vertebral endplate or facet joint. The clinical and laboratory data of these patients were retrieved from medical records.

**Results:** Out of 95 patients, 15 [15.8%; 95% confidence interval (CI), 9.4–25.0%] had a conventional CT evidence suggestive of axial gout. In these 15 patients, 12 (80%) had lumbar spine involvement (95% CI, 51.4–94.7%). Fifteen patients had erosions of the vertebral column, and two presented with tophi that exhibited erosive changes of the facet joints. The presence of axial gout was not associated with the patients' age, duration of gout, laboratory findings, inflammatory back pain symptoms, identification of monosodium urate crystals in the peripheral joints, current use of urate-lowering drugs, hypertension, and end-stage renal disease; however, there was a significant association with the presence of diabetes (*P* = 0.008).

**Conclusions:** The frequency of axial deposition in Korean patients with gout and spinal symptoms was 15.8%, with the lumbar region being the most commonly involved section of the spine. In addition, diabetes was associated with evidence of axial gout on imaging.

## Introduction

Gout is the most common inflammatory arthritis and is caused by an accumulation of monosodium urate (MSU) crystals in the synovial fluid (1–3). The MSU crystals accumulate in the synovial fluid and form deposits on the cartilage and, potentially, every tissue of the body, including the axial skeleton, where the facet joints, spinous processes, intervertebral disk, or sacroiliac joints may have MSU deposits (1, 4, 5). Gout typically involves the peripheral joints of the appendicular skeleton. However, the axial skeleton is not spared, and axial gout, which is gout of the spinal column, has been diagnosed more frequently with increased physician awareness of its existence and increased incidence of hyperuricemia (6). However, axial gout is still under-recognized to spine physicians, although it may be a cause of axial and radicular pain or myelopathy.

A few studies using conventional computed tomography (CT) have demonstrated the prevalence, clinical manifestations, and risk factors of axial gout in Caucasian and African Americans (7–9). The classic CT findings of axial gout are intra-articular and juxta-articular osseous erosions with sclerotic margins and tophaceous deposit shown as juxta-articular soft tissue masses with an attenuation density greater than the surrounding muscle (8, 10). Recently, dual-energy CT (DECT) has been used to identify MSU deposits with high sensitivity (11). There are racial differences in the incidence of gout; however, there have been no reports describing the characteristics of axial gout in the Asian population (9, 12). Therefore, the aim of this study was to describe the frequency of axial gout using CT and clinical characteristics in a Korean population. Furthermore, we present a clinical case to demonstrate the feasibility of using DECT to diagnose axial gout.

## Methods

### Subjects

We retrospectively selected Korean patients who visited our inpatient or outpatient spine centers owing to cervical or lumbar spine problems from March 2012 to February 2016. These patients had previously been diagnosed with gout and had CT images of the vertebral column, including spinal and abdominal CT. In our hospital, abdominal CT includes the lumbar spine. If both spinal and internal organ CT is available, the evaluation is based on spinal CT. A diagnosis of gout was made if a history of gout was documented in the medical records from the spine center clinic. In addition, documentation of gout in the medical records of other departments, such as rheumatology or nephrology, was acceptable for diagnosis. This research was approved by the Institutional Review Board (IRB No. 2016-06-049-001).

The clinical and laboratory data of the included patients were retrieved from medical record reviews and included age, sex, duration of disease, and the presence of (i) hypertension, (ii) diabetes, and (iii) end-stage renal disease. In addition, the main symptoms at the visit to the spine center (axial pain, radicular pain, myelopathic symptoms, or neurogenic claudication due to lumbar stenosis), identification of MSU crystals in the peripheral joints, current use of urate-lowering drugs (allopurinol, febuxostat, etc.), and serum uric acid and C-reactive protein levels were included. The characteristic of axial pain was classified as inflammatory or mechanical. Inflammatory back pain was considered if the patient had back pain for more than 3 months, suffered from back pain at night, and if the pain improved with exercise. These criteria were adapted and modified from the criteria of the Assessment of SpondyloArthritis International Society (13). The main symptom of myeloradicular pain or claudication combined with axial pain was considered as mechanical back pain.

### Imaging Studies

**Figure 1:**
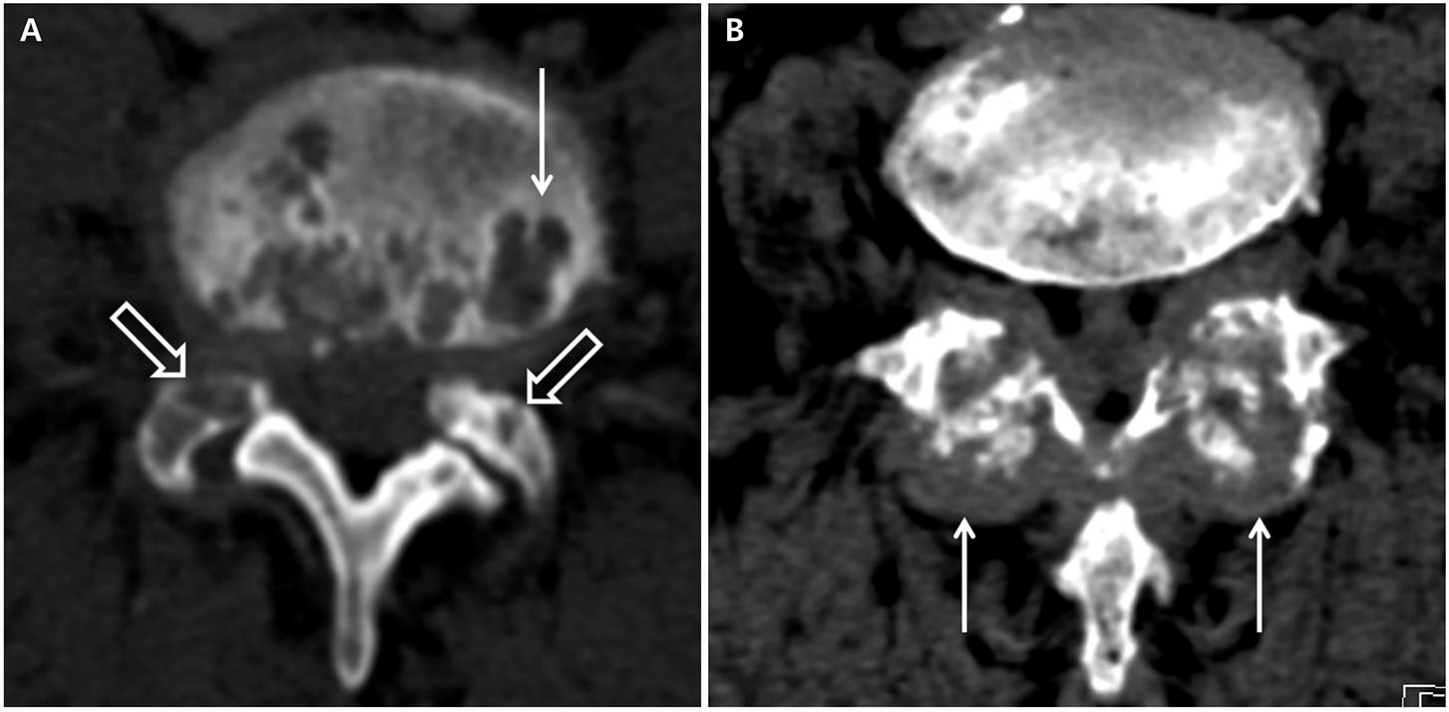
Computed tomography suggestive of axial gout. **(A)** Erosive changes with sclerotic margins in the vertebral body (white arrow) and facet joints (white hollow arrows). **(B)** Tophi in the facet joints (white arrows).

The presence of axial deposition (axial gout) was based on conventional CT imaging, which was reviewed by a single radiologist to determine whether the lesions were axial gout. Axial gout was defined by the presence of any of the following findings on spinal CT: erosive changes with sclerotic margins in the vertebral body and endplates or facet joints or tophi (lobular juxta-articular masses with an attenuation density greater than the surrounding muscle) (Figure 1) (8, 10). If, however, the intervertebral disc vacuum phenomenon, traction osteophytes, claw osteophytes, or wraparound bumper osteophytes on conventional CT were dominant, degenerative spondylosis was considered rather than axial gout (14). Axial pseudogout was considered if the nodular or ovoid calcified lesions continuous with the lamina, disc, posterior longitudinal ligament, or facet joint were prominent on conventional CT (15). Destructive spondyloarthropathy was considered if severe narrowing of the intervertebral disc space and erosions and cysts of the adjacent vertebral endplates with minimal osteophyte formation were visible on conventional CT in hemodialysis patients (16).

### Analyses

Our primary analysis was a retrospective, cross-sectional survey of the frequency of axial gout in a symptomatic Korean population attending a spine center clinic. The secondary analyses included the topographic spinal location of axial gout, its radiologic features, and the factors possibly associated with it.

### Statistical Methods

Chi-square test, Fisher's exact test, and Mann–Whitney *U*-test were used to identify significant variables in the comparison of proportion and distribution between axial gout and non-axial gout group. *P* < 0.05 (two-sided) was considered as statistically significant. All statistical analyses were performed using SPSS 18.0 software for Windows® (SPSS Inc., Chicago, IL, USA).

## Results

### Demographic Characteristics

In total, 95 patients (68 men and 27 women) were enrolled and included in this study. Of the 95 patients with gout, 35 were proven by the identification of MSU crystals in the peripheral joints and 60 were clinically diagnosed. The demographic and clinical characteristics are presented in Table 1. The patients' mean age was 66.0 ± 13.2 years. The mean duration of gouty arthritis was 6.3 ± 6.0 years (range, 1–29 years).

**Table 1:** Characteristics of the enrolled patients (*n* = 95).

**Variables**	**Number**
Age	66.0 ± 13.2
Sex (male/female)	68:27
Monosodium urate crystal—proven in the peripheral joints, *n*	35
Duration of disease, years	6.3 ± 6.0
Serum uric acid, mg/dl	7.7 ± 2.4
Diabetes, *n*	31
Hypertension, *n*	64
Main Symptomatic Lesions at Visit to the Spine Center Based on Clinical Findings	
Cervical lesion (axial pain or myeloradicular pain)	29
Lumbosacral lesion (axial pain, radicular pain, or claudication)	50
Cervical and lumbar lesions	16

### Clinical and Radiographic Characteristics of Axial Gout

The vertebral regions covered by CT and the results for each vertebral level are shown in Table 2. Forty-five patients were evaluated by cervical spine CT to determine if axial gout of the cervical spine was present, and 66 patients were evaluated by lumbar spine (*n* = 60) and abdominal (*n* = 6) CT to determine if axial gout of the lumbar spine was present. As shown in Table 3, 15 [15.8%; 95% confidence interval (CI), 9.4–25.0%] of the 95 patients had conventional CT evidence suggestive of axial gout with erosive changes or tophi with erosions, while two patients had both cervical and lumbar lesions. Thus, a total of 17 vertebral lesions (cervical or lumbar lesions) in 15 patients were detected as axial gout on conventional CT. The detection rate of cervical and lumbar spine involvement was 11.1% (95% CI, 4.2–24.9%) and 18.2% (95% CI, 10.1–30.0%), respectively. Furthermore, in 12 patients (80%; 95% CI, 51.4–94.7%) of the 15 patients, axial gout affected the lumbar spine, whereas five (33.3%; 95% CI, 13.0–61.3%) of these 15 patients had cervical spine involvement (Table 3).

**Table 2:** The vertebrae level covered by CT scan and the result of axial gout changes for each vertebrae level.

**Vertebrae level**	**Number of vertebrae scanned by CT**	**Scan method**	**Axial gout changes (%)**
Cervical spine	45	Cervical spine CT 45	5 (11.1%, 95% CI, 4.2–24.9%)
Lumbar spine	66	Lumbar spine CT 60 Abdomen CT 6	12 (18.2%, 95% CI, 10.1–30.0%)

**Table 3:** Characteristics of axial gout (*n* = 15).

**Variables**	**Number**
Overall number of patients	15
Overall numbers of lesions by vertebral level (cervical or lumbar)	17
Sex (male/female)	11/4
Duration of Gout	
Over 10 years	5
Between 2 and 10 years	7
<2 years	3
Topographic Location	
Cervical spine involvement	5
Lumbar spine involvement	12
Main Clinical Manifestations According to Lesions	
Cervical spine	
Axial pain	3
Myeloradiculopathy	2
Lumbar spine	
Axial pain	8
Spinal stenosis or radiculopathy	4
Radiologic Abnormalities	
Erosion	17
Vertebral endplate only	2 (cervical, 2)
Facet joints or spinous process	5 (lumbar, 5)
Vertebral endplate and facetjoint or spinous process	10 (cervical, 3; lumbar, 7)
Tophi with erosion	2 (lumbar, 2)

The radiologic findings revealed that all 17 vertebral lesions (cervical or lumbar lesions) had erosive changes, and two lumbar spine lesions had tophi with erosive changes of the facet joints. Out of these 17 erosive changes, two lesions were localized only to the vertebral endplate, five were found only in the facet joints or spinous processes, and 10 were observed both in the vertebral endplate and the facet joint or spinous processes.

Out of the 15 patients with axial gout on conventional CT, three underwent CT-guided biopsy because it was difficult to clinically differentiate axial gout from infectious or degenerative spondylosis. Subsequently, they were diagnosed with axial gout by identifying negatively birefringent MSU crystals on polarizing microscopy and were thus included in the study.

Axial neck pain (three patients) or myeloradiculopathy (two patients) were the main clinical findings of patients with axial gout of the cervical spine. Axial gout of the lumbar spine was related to axial back pain (eight patients) and spinal stenosis or radiculopathy (four patients).

Based on the classification of axial pain, in the axial gout group (15 patients), six (40.0%; 95% CI, 17.5–67.1%) had inflammatory back pain and nine (60.0%; 95% CI, 32.9–82.5%) had mechanical back pain. In the non-axial gout group (80 patients), 17 (21.6%; 95% CI, 13.2–32.1%) had inflammatory back pain and 63 (78.9%; 95% CI, 67.9–86.8%) had mechanical back pain.

### Comparison of the Presence of Axial Gout With Clinical Factors

There were no associations of the presence of axial gout with age (*P* = 0.145), duration of gout (*P* = 0.570), serum C-reactive protein (*P* = 0.944), and uric acid level (*P* = 0.405), characteristics of axial pain (*P* = 0*.185*), identification of MSU crystals in the peripheral joints (*P* = 0.159), current use of urate-lowering drugs (*P* = 0.416), the presence of hypertension (*P* = 0.453), and end-stage renal disease (*P* =0.284). However, axial gout was significantly associated with the presence of diabetes (*P* = 0.008) (Table 4).

**Table 4:** Comparison of factors between axial gout and non-axial gout.

**Variables**	**Axial gout (*n* = 15)**	**Non-axial gout (*n* = 80)**	***P***
Age	67.1 ± 12.1	65.0 ± 13.3	0.545
Duration of disease	6.6 ± 5.0	6.2 ± 6.2	0.570
C-reactive protein, mg/dl	1.4 ± 2.2	1.5 ± 2.6	0.944
Serum uric acid, mg/dl	7.8 ± 2.3	7.7 ± 2.5	0.405
Monosodium urate crystal—proven in the peripheral joints, *n* (%)	8 (53.3)	27 (33.8)	0.159
Current urate-lowering drug, *n* (%)	11 (73.3)	53 (66.3)	0.416
Inflammatory back pain, *n* (%)	8 (53.3)	17 (21.3)	0.185
End-stage renal disease, *n* (%)	5 (31.3)	11 (13.8)	0.284
Diabetes, *n* (%)	10 (66.7)	21 (26.3)	0.008
Hypertension, *n* (%)	10 (66.7)	54 (67.5)	0.453

### Illustrative Case

**Figure 2:**
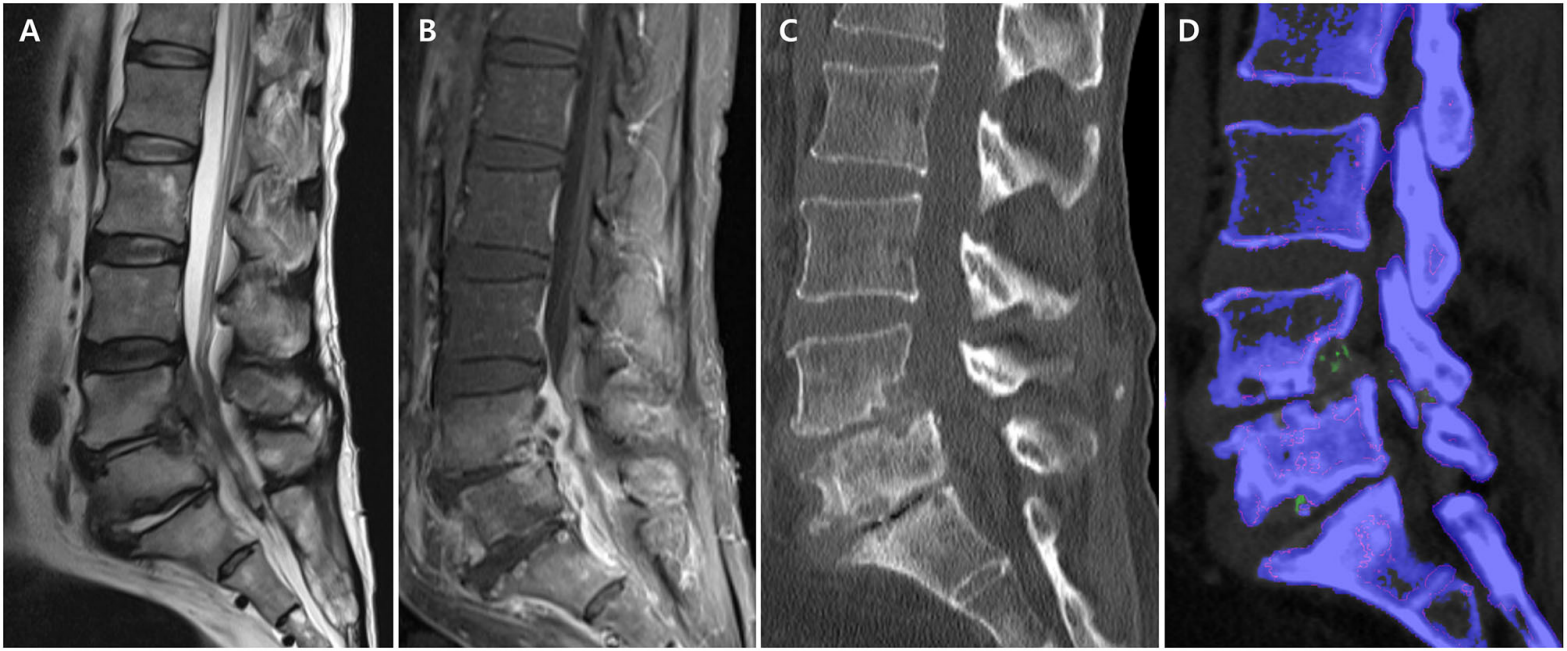
Lumbar spine magnetic resonance imaging and computed tomography (CT) in axial gout. **(A)** Increased signal intensity on T2WI of L4-5 and L5-S1 intervertebral disc and erosive changes on the posterior cortices and endplates of L4-L5-S1 vertebra. **(B)** Enhancement of epidural space on T1WI. **(C)** Erosive changes in L4-5 and L5-S1 endplates on conventional CT. **(D)** Monosodium urate deposits (green) in the erosive foci of endplate on dual-energy CT.

A 54-years-old Korean male patient with a 5-years history of gout and chronic lower back pain visited our emergency room with severe back pain and right-sided buttock pain for 1 week. He had taken urate-lowering agents irregularly and occasionally received an oral steroid. The patient complained of difficulty in standing from when he was sitting due to back pain but reported relief of the pain with activity. On physical examination, he had limited motion of the lumbar spine and focal tenderness over the lower back area. He had a mild fever (37.9°C). Motor weakness and sensory abnormalities were not found on both lower extremities. Blood tests showed a white blood cell count of 13,900/mm^3^, erythrocyte sedimentation rate of 94 mm/h, and C-reactive protein of 7.72 mg/dl. Lumbar spine magnetic resonance imaging (MRI) demonstrated increased signal intensity on T2WI of L4-5 and L5-S1 intervertebral disks, erosive changes on the posterior cortices and endplates of L4-L5-S1 vertebra, and enhancement of the epidural space (Figures 2A,B). Based on the physical examination, laboratory findings, and MRI, we made a provisional diagnosis of acute pyogenic spondylodiscitis and started treatment with broad-spectrum intravenous antibiotics. On day 5 of antibiotic treatment, he complained of severe right knee and ankle pain and swelling of the joints. The knee joint fluid was analyzed using polarized microscopy, and negatively birefringent crystals were identified. A lumbar spine DECT was performed to investigate the possibility of axial gout. Images showed MSU signal intensity (green) on erosive foci of the endplates and on the facet joints (Figures 2C,D). A CT-guided biopsy for the erosive lesions was performed, and MSU crystals from the spine biopsy were identified using polarized microscopy. We considered his symptoms as axial gout instead of pyogenic arthritis and spondylodiscitis. His symptoms dramatically improved with non-steroidal anti-inflammatory medicines combined with a short course of prednisolone. After resolution of the gout flare of the spine, he occasionally complained of intermittent mild lower back pain.

## Discussion

This retrospective study aimed to describe the frequency and possible associated factors of axial deposition (axial gout) among Korean patients with gout and spinal symptoms in a tertiary spine center. In addition, we presented an illustrative case study of axial gout that demonstrated the feasibility of utilizing DECT to diagnose axial gout. In our study, 15 out of 95 (15.8%) patients with gout and spinal symptoms had CT evidence suggestive of axial gout. The lumbar spine (12 patients, 80%) was the commonly involved region. In addition, diabetes was associated with axial deposition in patients with gout.

Several studies have investigated the clinical and the radiologic findings of axial gout (5–10, 17). The prevalence of axial gout is varied based on the design and the demographics of the study population, with a range of 14–35% in gout patients (7–9). The most commonly studied population was the African cohort, which was more predisposed to developing gout than the Caucasian cohort. Individuals of African descent are less likely to be treated with urate-lowering agents and typically present with higher rates of independent comorbidities associated with gout, such as hypertension, obesity, and renal impairment (8, 9, 12). These comorbidities may explain why patients of African descent present with more severe variants of gout, such as axial gout. A previous study reported that 17 (35%) of the 48 subjects, 42 of whom were African, had CT findings suggestive of axial gout (9). Coincidentally, this study suggested that the frequency of axial deposition (15.8%) in Korean patients, with gout and spine problems, was comparable to the results of populations of African descent. However, because of several limitations of this study, such as the retrospective study design and the possibility of selection bias, further prospective studies will be necessary to determine if race influences the development of axial gout.

We expected that the duration of gout would be associated with an increased likelihood of the presence of axial gout. This study did not demonstrate that the duration of gout was a possible association factor for axial deposition in patients with gout. The concept that peripheral gout precedes axial gout has been generally accepted. However, a recent review reported that axial gout may be the first clinical manifestation of gout in 24.8% of patients (18). In this study, three (20%) out of 15 patients with axial gout had disease durations of <2 years. This result suggests that axial deposition can develop irrespective of the presence or the absence of gout. Furthermore, there was a significant association of axial gout with diabetes. It is not clear why diabetes is a possible associated factor of axial deposition in patients with gout, although it is well-recognized that diabetes is a risk factor for gout and *vice versa* (19–21).

Although the demonstration of MSU crystals in tissue samples is the gold standard for diagnosing gout, previous studies suggested that CT is a useful imaging modality in the initial diagnosis of axial gout (5, 7–10, 18). Hongli et al. raised a question on whether the gout-suggestive features on CT are specific for axial gout or gouty arthritis, especially for abnormal bony neoformations of the facet joints and intervertebral disk (22). Thus, these abnormal bony neoformations were not considered as being a suggestive feature of axial gout in the CT in the present study. Even in older patients without gout, the presence of subchondral erosions, cysts, and sclerosis of the facet joints and endplates was not uncommon, and these findings are similar to the axial gout-suggestive features seen on CT (23). Rheumatoid arthritis, infective arthritis, or metastasis can present with erosive lesions of the spine (24, 25). In addition, it is difficult to identify the causes of radiologic-proven destructive spondyloarthropathy in patients with long-term hemodialysis (25, 26). Thus, a survey of axial gout based on CT findings may overestimate its frequency. Nevertheless, we paid special attention to differentiate gout-induced changes from degenerative findings or other spondylodiscitis conditions that may mimic gout. We determined that erosive lesions of the endplates or facet joints without disc calcification in contiguous and non-contiguous multilevel preferred to axial gout. Further studies are necessary to describe the characteristics of axial gout-specific erosive lesions on CT in larger samples.

Percutaneous spinal biopsy for the confirmation of axial gout is an invasive and risky procedure and has less sensitivity (17, 27). Consequently, high-resolution CT has replaced spinal biopsy in the diagnosis of axial gout. Nevertheless, conventional CT is limited in that it can only be used to visualize secondary bony or soft tissue changes and not direct MSU deposits. Recently, DECT has been increasingly used to identify MSU deposits in the appendicular skeleton as a highly sensitive and specific radiologic tool (11). The diagnostic use of DECT in axial gout has been rarely reported (28, 29). In our illustrative case study with histological confirmation, the identification of MSU crystals within the foci of erosive lesions on DECT strongly suggested axial gout. Further studies are required to determine whether DECT can be useful in diagnosing axial gout in a larger sample.

There are several limitations to our study. First, the cohort studied was selected because it was composed of patients who presented to a spine clinic, had concomitant gout, and had available CT imaging results. Therefore, the results cannot be generalized to the general population. Second, because up to 20% of clinically diagnosed gout can be incorrect, the proportion of MSU crystal-proven gout in this study was small (30). Third, this study was retrospective in nature. Fourth, the number of enrolled patients was small. Thus, our results did not confirm a true correlation between various possible risk factors and axial gout. Fifth, not all cases of axial gout diagnosed by CT findings were confirmed by histological examination. Sixth, different spinal segments were not equally examined because patients with low back pain or radiating pain of the lower extremities and those with neurogenic claudication visited our clinic more frequently. Lastly, although we made an effort to differentiate gout-induced bony or soft tissue changes from other mimicking lesions, CT findings suggestive of gout occasionally were not absolutely certain.

## Conclusion

The frequency of axial deposition (axial gout) in Korean patients with gout and spinal symptoms included in this study was 15.8% (15 of 95 patients), with the lumbar spine being the commonly involved section of the spine. Diabetes, rather than the duration of gout, was associated with the presence of axial deposition in patients with gout. An illustrative case suggested that DECT may be a useful tool in the diagnosis of axial gout.

## Data Availability Statement

The datasets generated for this study are available on request to the corresponding author.

## Ethics Statement

The study involving human participants was reviewed and approved by Institutional Review Board of Dongsan Medical Center. Informed consent was exempted as CT scans were performed as a diagnostic procedure with benefits for the patients.

## Author Contributions

E-SS and DK designed, acquired, and analyzed the data. E-SS, H-JJ, and DK interpreted the data and revised the manuscript draft. All authors have read and agreed to the published version of the manuscript.

## Conflict of Interest

The authors declare that the research was conducted in the absence of any commercial or financial relationships that could be construed as a potential conflict of interest.
